# Incorporation of Carbon Nanofillers Tunes Mechanical and Electrical Percolation in PHBV:PLA Blends

**DOI:** 10.3390/polym10121371

**Published:** 2018-12-11

**Authors:** Jesse Arroyo, Cecily Ryan

**Affiliations:** Mechanical and Industrial Engineering Department, Montana State University, P. O. Box 173800, Bozeman, MT 59717, USA; JesseArroyo@montana.edu

**Keywords:** biodegradable polymers, biopolymer, nanofiller, partitioning, localization, biocarbon, biochar, percolation, phase separation, conductivity

## Abstract

Biobased fillers, such as bio-derived cellulose, lignin byproducts, and biochar, can be used to modify the thermal, mechanical, and electrical properties of polymer composites. Biochar (BioC), in particular, is of interest for enhancing thermal and electrical conductivities in composites, and can potentially serve as a bio-derived graphitic carbon alternative for certain composite applications. In this work, we investigate a blended biopolymer system: poly(lactic acid) (PLA)/poly(hydroxybutyrate-*co*-hydroxyvalerate) (PHBV), and addition of carbon black (CB), a commonly used functional filler as a comparison for Kraft lignin-derived BioC. We present calculations and experimental results for phase-separation and nanofiller phase affinity in this system, indicating that the CB localizes in the PHBV phase of the immiscible PHBV:PLA blends. The addition of BioC led to a deleterious reaction with the biopolymers, as indicated by blend morphology, differential scanning calorimetry showing significant melting peak reduction for the PLA phase, and a reduction in melt viscosity. For the CB nanofilled composites, electrical conductivity and dynamic mechanical analysis supported the ability to use phase separation in these blends to tune the percolation of mechanical and electrical properties, with a minimum percolation threshold found for the 80:20 blends of 1.6 wt.% CB. At 2% BioC (approximately the percolation threshold for CB), the 80:20 BioC nanocomposites had a resistance of 3.43 × 10${}^{8}$ Ω as compared to 2.99 × 10${}^{8}$ Ω for the CB, indicating that BioC could potentially perform comparably to CB as a conductive nanofiller if the processing challenges can be overcome for higher BioC loadings.

## 1. Introduction

Increasing concern for the environment and volatile petroleum prices has led to growth of bio-based and biodegradable materials as alternatives to petroleum derived plastics [1]. Plastics from bio-derived sources, or bioplastics, can be processed from a variety of feedstock including raw and refined plant sources and methane gas from biological degradation processes [2]. One class of bioplastics, poly(hydroxyalkanoates) (PHAs), such as poly(hydroxybutyrate) (PHB) and its industrially-produced copolymer poly(hydroxybutyrate-*co*-hydroxyvalerate) (PHBV), is synthesized by microorganisms as a storage polymer and can be harvested to produce a usable plastic [3]. PHBV has mechanical properties most similar to polypropylene, however thermal processing of PHBV is challenging due the proximity of the thermal decomposition temperature to the melting temperature [4,5]. Poly(lactic acid) (PLA) is another important bioplastic, that can be produced through renewable resources, and has mechanical properties most similar to that of polystyrene [5]. Alone, both bioplastics are brittle, with relatively poor impact strength and low thermal degradation temperatures. The toughness and processability of these bioplastics can be improved through multiphase blends of PHBV and PLA, resulting in attractive material properties not obtainable in the neat biopolymers [2,6].

One growing application space for bioplastics is additive manufacturing, and an emergent area of this space is conductive filament. Conductive filament is used in the rapid prototyping and production of electrically conductive components on a variety of 3D printers [7,8]. This production method enables various applications from low cost sensors to conductive traces, branching into electromagnetic and radio frequency shielding [9,10]. Typically, conductivity in thermoplastics is achieved through the addition of a conductive filler, such as silver nanoparticles, carbon black nanofillers, or graphene [10,11]. In addition to electrical conductivity, these value-added composites can reduce cost and weight, add color, provide anti-static potential at low volume percent, and enhance the mechanical and thermal properties over that of the neat polymer [12]. Of these fillers, the majority of conductive filaments are produced with carbon black (CB), a commercially available petroleum-derived filler.

Though CB offers many positive benefits to polymer blends, when designing biobased composites having a renewable source for and considering the fate of filler materials is also important. Biochar (BioC) carries many of the same benefits as CB, but comes from renewable plant-derived sources and, as a widely applied agricultural amendment, is compatible with the bioplastic’s ability to biodegrade. This bio-sourced form of carbon is produced in a similar way to charcoal. Thermal decomposition of biomass in the absence of oxygen results in roughly 35% syngas, 30% bio-oil, and 35% BioC [13]. The electrical conductivity of lignin sourced BioC has been shown to improve with pyrolysis at high treatment temperatures (over 800 °C) [14,15,16].

The bulk of the work exploring conductive nanocomposites, including with polymer blends, consists of polymers mixed with CB, graphene, carbon nanotubes (CNTs), or other graphite fillers [7,8,17,18,19]. The conductivity of PLA-based composites has been successfully modified with CNTs [20,21], graphene [22,23,24,25], and CB [26,27]. Conductive filament made from PLA and conductive fillers (CB, graphene) is commercially available [28,29]. Fewer conductivity studies have been done with PHB and PHBV-based carbon nanocomposites; this work has been primarily with CNTs and graphene or graphene oxide, with conductivities in the range of ∼0.1 S/m to 30 S/m with loadings above the percolation threshold [30,31,32].

Studies of conductive polymer blends have shown that nanofillers lower the percolation threshold of the blend over that of nanofiller incorporated into a single polymer, due to the ability to reside in either the minor or major phase, or the interfacial region [19]. This partitioning of the nanofiller produces higher conductivity at lower weight percent filler than in their non-blended counterparts. Phase-separation has been used to control nanofiller localization in blended PLA composites [20,21,22,33]. Differences in nanofiller aspect ratio contribute to the phase-localization behavior in addition to phase separation in these composite blends [27,34,35]. The studies of BioC involve particulate in a single polymer, conductivity of non-incorporated monolithic BioC, or large (microns to millimeters) fillers in a polymer blend [12,14,36,37].

This study provides a novel investigation into the localization of CB and BioC nanofiller in biopolymer blends, and the resultant impact on mechanical and electrical properties of nanocomposites of interest for 3D printing. The focus is to investigate: (i) Nanofiller localization and resultant morphology in a blended biopolymer system, PHBV:PLA, (ii) electrical and mechanical percolation of the nanofilled composites, and (iii) the impact of the two nanofillers on processing and melt rheology. To elucidate how nanofiller localization would be expected to occur in the blends, we predict the interfacial energies of the blends using contact angle measurements and calculations of surface tension and verify these predictions using field emission scanning electron microscopy (FE-SEM). We then use impedance spectroscopy, Raman spectroscopy, and dynamic mechanical analysis (DMA) to evaluate the electrical and mechanical properties of the nanocomposites and calculate the percolation threshold in blended and non-blended systems. We use melt rheology during compounding and differential scanning calorimetry (DSC) to evaluate the impact of nanofiller addition during processing and how these interactions impact blend microstructure, thermal stability, and processability.

## 2. Theory

Polymer blend phase separation and incorporation of nanofillers can be described through the use of the Owens-Wendt theory [38]. The thermodynamics of phase behavior of polymers in a blend is governed by the surface tension of the polymers. The surface tension is comprised of polar and dispersive components, and is typically measured using the contact angle between the polymer surface and liquids with known polar and dispersive values. The Owens-Wendt theory combines the Goods Equation (1) with the Young’s Equation (2) to create the linear form (3) [38]:

(1) $$\gamma_{sl}=\gamma_{s}+\gamma_{l}-2\sqrt{\gamma_{l}^{d}\gamma_{s}^{d}}-2\sqrt{\gamma_{l}^{p}\gamma_{s}^{p}}$$

(2) $$\gamma_{s}=\gamma_{sl}+\gamma_{l}cos\left(\theta \right)$$

(3) $$\frac{\gamma_{l}cos\left(\theta +1\right)}{2\sqrt{\gamma_{l}^{d}}}=\gamma_{s}^{p}\frac{\sqrt{\gamma_{l}^{p}}}{\sqrt{\gamma_{l}^{d}}}+\sqrt{\gamma_{s}^{d}}$$

Substituting into the linear form (y=mx+b) gives:

(4) $$y=\frac{\gamma_{l}cos\left(\theta +1\right)}{2\sqrt{\gamma_{l}^{d}}}$$

(5) $$m=\gamma_{s}^{p}$$

(6) $$x=\frac{\sqrt{\gamma_{l}^{p}}}{\sqrt{\gamma_{l}^{d}}}$$

(7) $$b=\sqrt{\gamma_{s}^{d}}$$

where $\gamma_{l}$ is the overall surface tension of the wetting liquid, $\gamma_{s}$ is the overall surface energy of the solid, the polar and dispersive components are represented by $\gamma_{s}^{p}$,$\gamma_{l}^{p}$ and $\gamma_{s}^{d}$, $\gamma_{l}^{d}$ respectively, $\gamma_{sl}$ represents the interfacial tension between the solid and the liquid, and θ is the contact angle between the liquid and the solid. A solid’s unknown polar and dispersive components ($\gamma_{s}^{p}$,$\gamma_{s}^{d}$) are calculated using contact angles with liquids of a known polar and dispersive component ($\gamma_{l}^{p}$,$\gamma_{l}^{d}$). This calculation is done by plotting contact angle data (x,y: Equations (4) and (6)) and using a line of best fit to determine a slope and *y*-intercept [39]. This form of the Harmonic Mean Method requires a minimum of two liquids for which surface tension data is well known to develop a best fit line.

In general, binary polymer blends exhibit either a blended morphology representative of miscibility, or they may exhibit a sea-island structure representative of an immiscible blend. For binary polymer composites, there are typically three potential locations of the nanofiller: It may exist in the major phase, the minor phase, or in the interfacial region between the two. The interfacial tension between the blended polymers and the nanoparticulates was calculated using the Harmonic Mean Equation (8), and used to predict the morphology of the system [39].

(8) $$\gamma_{ij}=\gamma_{i}+\gamma_{j}-\frac{4\gamma_{i}^{d}\gamma_{j}^{d}}{\gamma_{i}^{d}+\gamma_{j}^{d}}-\frac{4\gamma_{i}^{p}\gamma_{j}^{p}}{\gamma_{i}^{p}+\gamma_{j}^{p}}$$

To determine miscibility of the polymer blend, the spreading coefficient $\lambda_{ij}$ was calculated for phase *i* on phase *j* (9). A positive $\lambda_{ij}$ indicates that polymer *i* will spread and is miscible on *j*, while a negative number indicates immiscibility between *i* on *j* [39].

(9) $$\lambda_{ij}=\gamma_{j}-\gamma_{i}-\gamma_{ij}$$

The localization of the nanofiller in the blend can be predicted by determining the wetting coefficient of the polymer on the particulate, $\omega_{ij}$. This coefficient is given in Equation (10), where $\gamma_{i,NF}$ represents the interfacial tension of the nanofiller on polymer *i* [39].

(10) $$\omega_{ij}=\frac{\gamma_{i,NF}-\gamma_{j,NF}}{\gamma_{ij}}$$

By this definition, if $\omega_{ij}$ is greater than 1 the particulate will localize in the *j* phase of the polymer blend. In turn, if $\omega_{ij}$ is less than −1 it will localize in the *i* phase and finally if $\omega_{ij}$ is between 1 and −1 the particulate will localize in the interfacial region between the blends.

The contact angle between the polymers during molten flow can be characterized by Equation (11) and used to predict the shape of the minor phase in the major [39].

(11) $$\theta_{ij}=cos^{-1}\left(\frac{\gamma_{j}-\gamma_{ij}}{\gamma_{i}}\right)$$

## 3. Materials and Methods

### 3.1. Materials

Commercially available PHBV (ENMAT Y 1000p, >98% purity) in pellet form was provided by Tianan (Nigbo City, China). PLA (2003D), also in pellet form, was supplied by Nature Works (Minnetonka, MN, USA). Powdered CB (Vulcan XCMAX22) with a density of 0.19 g/cm${}^{3}$ was provided by Cabot Chemical Corporation (Boston, MA, USA). Kraft lignin, with a density of 1.3 g/cm${}^{3}$ at 25 °C, was purchased from Sigma-Aldrich, St. Louis, MO, USA and used to produce the BioC. All materials were stored in a desiccator prior to composite fabrication.

### 3.2. BioC Production

BioC was produced through slow pyrolysis of ball milled kraft lignin. Powdered kraft lignin (20 g) was milled with zirconia media for 24 h at 60 rpm. The media were removed and the milled lignin was stored at 105 °C to remove moisture. Prior to pyrolysis in a tube furnace, nitrogen gas was purged through the tube at 0.95 CCM for 15 min to establish an oxygen free environment. After the initial purging, nitrogen flow was reduced to 0.55 CCM and heating began at 10 °C/min to 750 °C. After one hour at 750 °C the temperature was ramped to 950 °C at a rate of at 10 °C/min and held for an hour. The sample was then allowed to cool to room temperature while still under nitrogen flow. Post pyrolysis, samples were stored at 105 °C.

### 3.3. Composite Fabrication

Composites were prepared by melt compounding in a Thermo Fisher Scientific HAAKE Minilab II dual screw extruder at 50 rpm and 190 °C for 5 min. During this time, rheology data were collected and viscosity was calculated from Minilab outputs as shown in Appendix B. After mixing, blends were extruded into a Thermo Fisher Scientific Minijet Pro injection molder. Initial injection pressure was 600 bar for 10 s followed by 450 bar for 60 s. Injection temperatures were 190 °C in the gun and 60 °C in the mold, as established through prior optimization. All composites were injected into a DMA sample mold (Thermo Fisher Scientific, Waltham, MA USA, Part # 557-2295) with dimensions of 60 × 10 × 1 mm${}^{3}$. Table 1 gives the blend ratios used for the PHBV:PLA blends and blends with nanofillers. CB nanofilled blends were made with all PHBV:PLA blend ratios, while samples of BioC were produced as feasible due to viscosity challenges during processing. Nanofiller was measured as a weight fraction of the total polymer blend.

### 3.4. Characterization

Interfacial parameters, polymer blend morphology, moduli, thermo-mechanical, and electrical properties were evaluated by video contact analysis, FE-SEM, DMA, DSC, Raman spectroscopy, and 4-point probe impedance spectroscopy measurements.

#### 3.4.1. Contact Angle Analysis

To better understand the interactions of the polymer blends during mixing, polar and dispersive components of polymer surface tensions were calculated by measuring contact angles with deionized water and diiodomethane (MI). Contact angles were measured using a video contact angle system with drop sizes of 2.45 ± 0.5 μL and a minimum of 5 measurements. Angles were divided into their dispersive and polar components using the Owens-Wendt relationship as described in Section 2 (Equations (1)–(3)). Table 2 shows the known dispersive and polar components of water and MI used as the contact liquid. Matlab code was developed to analyze the surface tensions and predict interfacial interactions, phase separation, and nanofiller localization in the polymer blends and nanofilled composites [40].

It is particularly difficult to consistently measure contact angles for nanofillers in order to determine surface energies. Instead, alternate methods such as absorption and heat of immersion are standard techniques and have been explored in previous studies which provided the literature values for CB used in this study [41,42].

#### 3.4.2. Dynamic Mechanical Analysis

Dynamic mechanical measurements were conducted on a TA instruments Q800 DMA. A multi frequency-strain experiment in the 3-pt bending configuration was run at a frequency of 1 Hz, amplitude of 20 μm, and a force track of 125%. The temperature was equilibrated at −40 °C for five minutes and increased to 150 °C at a constant rate of 5 °C/min. During the temperature ramp, storage modulus, loss modulus, and tanδ data were collected.

#### 3.4.3. Differential Scanning Calorimetry

A TA Instruments Discovery DSC (Serial Number DSCI-0220) was used to assess the impact of the nanofillers on the polymers and blends. The nitrogen flow rate was 50 mL/min, as optimized in previous work [43]. Samples were encapsulated in aluminum pans with a target sample weight of 5 mg ± 2 mg, and heated from −20 °C to 180 °C in the first heating cycle at a rate of 10 °C/min. After equilibrating to 190 °C, they were held at 190 °C for 2 min prior to cooling at 10 °C/min to −20 °C. The samples were then heated at 10 °C/min to 195 °C in the second and final heating cycle. The glass transition temperature ($T_{g}$) was taken to be the midpoint of the heat capacity change, the melting temperature ($T_{m}$) was measured as the minimum of the endothermic peak upon heating, the cold crystallization temperature ($T_{cc}$) was measured as the maximum of the exothermic peak (when present) upon heating, and the crystallization temperature ($T_{c}$) was taken as the maximum temperature of the exothermic peak upon cooling (in between the first and second heating cycles).

The percent crystallinity of the PHBV and PLA in the matrix, $\chi_{P}$, was determined using a modification to the standard equation for single phase composites [44]:

(12) $$\chi_{P}\;\left[\%\right]=\frac{\Delta H_{m}-\Delta H_{cc}}{\Delta H_{m}^{\circ }}\left(\frac{1}{W_{P}}\right)\cdot 100\%$$

where $\Delta H_{m}$ and $\Delta H_{cc}$ are the enthalpies of melting and cold crystallization measured upon heating, $W_{P}$ is the weight fraction for the PHBV or PLA, and $\Delta H_{m}^{\circ }$) is the reference value for 100% crystalline polymer: 146 J/g or 12.5 kJ/mole [45,46] for PHB and 93.7 J/g for PLA [47]. As a reference, a typical value for the crystallinity of annealed PHB samples measured by Barham was 86% et al. [45]. To convert between volume and weight percent, densities of 1.24 g/cm${}^{3}$ for PHBV and 1.25 g/cm${}^{3}$ for PLA were used. Sample density was measured using a Mettler Toledo XS205DU Excellence series analytical balance with the Mettler Toledo Density determination kit for Excellence XP/XS analytical balances. The measurement is a buoyancy technique based on the Archimedes’ principle.

To evaluate the effect of BioC on each of the individual biopolymers, DSC was used to evaluate neat PHBV and PLA compared with each of the polymers with BioC. BioC was added on top of each of the biopolymers prior to the first heating cycle. The melting endotherms of the neat polymers were compared to the endotherms of the first and second heating cycles in the BioC nanofilled biopolymers.

#### 3.4.4. Scanning Electron Microscopy

To obtain a cross section of the nanofilled blends, samples were cryo-fractured using liquid nitrogen. Examination of fracture surfaces though FE-SEM was conducted on a Supra 55VP System 2512 at 1 kV with an SE2 detector. Samples were uncoated. Nanofiller and matrix microstructure were characterized and the particle-matrix interfaces were imaged along with assessing potential localization of the nanofillers.

#### 3.4.5. Impedance Spectroscopy

The polymers’ resistivity, impedance, capacitance, and phase angle were measured using a Hioki 3522-50 LCR HiTester in the 4 point configuration at room temperature. Each data point is the average of 3 measurements taken from 0.1 Hz to 10${}^{5}$ Hz. The percolation threshold for the composites was calculated using a Sigmoidal–Boltzmann function [48]:

(13) $$\rho =\rho_{l}-\left(\frac{\rho_{l}-\rho_{u}}{1+e^{\frac{\varphi -\varphi_{c}}{\Delta \varphi }}}\right)$$

where ρ is the measured resistivity, $\rho_{l}$ is the lower limit for resistivity, $\rho_{u}$ is the upper limit for resistivity, φ is the percentage of CB in the blend, $\varphi_{c}$ is the percolation threshold, and Δφ is the slope in proximity to the percolation threshold. The data for resistivity from impedance spectroscopy at 10${}^{3}$ Hz were fitted using the curve fitting toolbox in Matlab R2018b.

#### 3.4.6. Raman Spectroscopy

A fully integrated high resolution Raman microscope for confocal Raman analysis, Horiba LabRam HR Evolution NIR, was used to evaluate CB, BioC, and nanofilled composites for graphitic content. The confocal microscope was used for optical images of the composite samples at 20× LWD and 50× LWD. During Raman spectral acquisition, Raman spectra were acquired at 50× LWD and 100×, the stigmatic spectrometer was used with a grating of 1800 gr/mm, and the 532 nm 100 mW laser at 1%. To reduce the impact of heating in the samples, the acquisition time was 3 s, and the spectra were accumulated for 3 acquisitions. Raman spectra were recorded between 1000 and 1800 cm${}^{-1}$, which corresponds to the spectral region that provides data on the microstructure of carbons giving a measure of the graphite band at 1530–1610 cm${}^{-1}$ (G) and the disorder-induced band at 1320–1370 cm${}^{-1}$ (D) [23,49,50]. The cftool in Matlab was used to fit gaussian exponentials to the CB and BioC peaks observed in this region to determine the peak locations for D and G peaks and the intensity ratio, IDIG [51].

## 4. Results and Discussion

### 4.1. Evaluation of Blend Morphology and Nanofiller Localization

#### 4.1.1. Predictions from Interfacial Tension

Contact angle analysis with water revealed that the two polymers exhibit hydrophilic characteristics as contact angles were below 90°. In addition, evaluation with MI showed that both PHBV and PLA exhibit dispersive dominate components (Table 3).

Table 4 shows predictions of miscibility of the polymer blends and the localization of the nanofiller within the blends made from Equations (9) and (10). A negative spreading coefficient between PHBV on PLA suggests that the PHBV phase is immiscible with the PLA phase. The tension between the CB and PHBV is significantly lower than that between the CB and PLA, suggesting that the CB will preferentially localize in the PHBV phase. This predicted separation is further supported by the negative wetting coefficient between CB on the PHBV major phase, and a positive wetting coefficient with the PLA major phase. These equations predict that for the blends studied here, PLA will form an immiscible structure within PHBV while CB will reside in the PHBV phase.

#### 4.1.2. Verification of Nanofiller Partitioning and Blend Morphology

**Figure 1:**
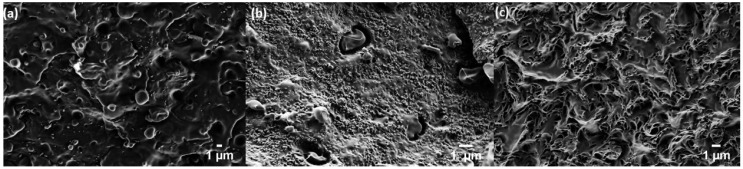
FE-SEM images of 80:20 PHBV:PLA with: (**a**) no nanofiller, (**b**) 6% CB, and (**c**) 6 wt.% BioC.

Figure 1 shows FE-SEM micrographs of an 80:20 PHBV:PLA blend with and without CB and BioC nanofiller. Immiscibility of PLA in PHBV is seen in its sea-island structure (Figure 1a), as predicted by the surface tension results. As CB is added to the blend (Figure 1b), it appears to localize in the major PHBV phase while the PLA phase is left absent of nanofiller. This morphology observed via FE-SEM shows a granularity characteristic of the CB nanofiller, and is comparable to other studies where SEM observation of carbon black localization was verified with transmission electron microscopy (TEM) [52,53]. Furthermore, the addition of CB does not alter the immiscibility of the two polymers. The effects of adding BioC to the blend can be seen in Figure 1c. In the blend with BioC, there is no visual distinction between the two polymer phases. Based on this observation and additional supporting evidence through mixing experiments, rheology, and DSC, we hypothesize that BioC reacts with the biopolymers, potentially preferentially depolymerizing the PLA phase. Previous studies have observed that reduced molecular weight PLA is miscible in PHBV and vice versa [54,55,56,57,58]. Therefore, the lack of observable phase separation in FE-SEM is potentially indicative of this molecular weight reduction. In summary, the partitioning of the CB nanofiller behaves as predicted. The interactions of the BioC with the polymer blend are more complex and are discussed in additional detail in Section 4.3.1 and Section 4.3.2.

**Figure 2:**
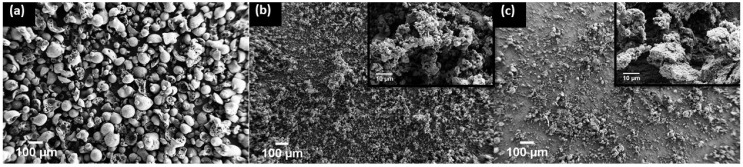
FE-SEM of (**a**) un-milled BioC, (**b**) milled BioC, and (**c**) CB shows that the size reduction of the BioC after milling is comparable to the particle size of the CB.

For the surface energy and partitioning predictions made in Section 4.1 to be accurate, the filler particle size should be smaller than the minor phase regions of the blend. The as-received lignin yielded BioC with an average particle size of approximately 100 μm, larger than the 1–3 μm domains of the PLA in the PHBV (Figure 1a,b and Figure 2a). The milled lignin yields BioC with a comparable particle size to that of the CB (Figure 2). This nano size enables the filler material to reside in either the minor or the major phase of the blend.

### 4.2. Electrical and Mechanical Percolation of Nanofillers

#### 4.2.1. Impedance Spectroscopy of Polymer Blends

**Figure 3:**
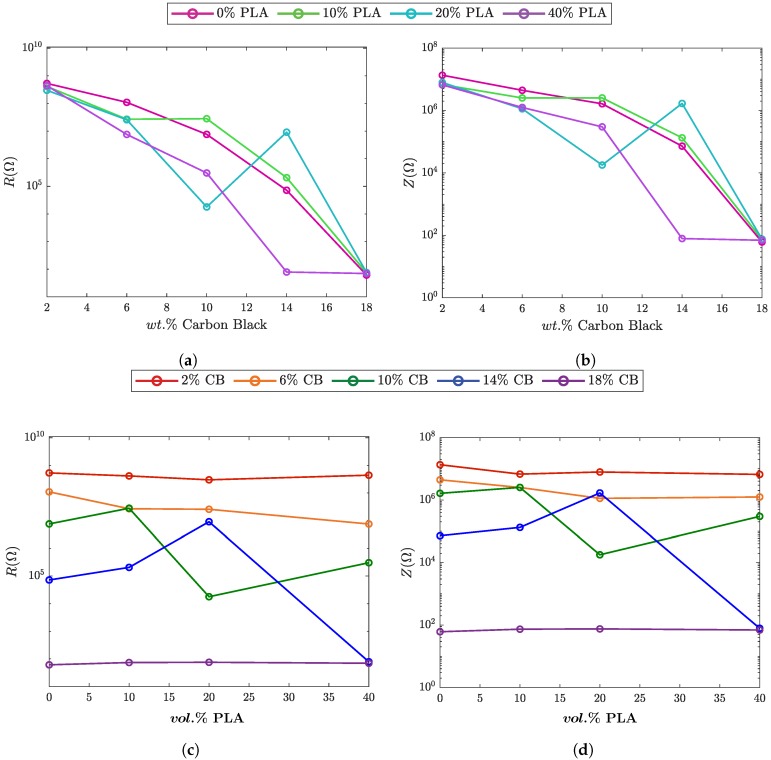
Four point impedance spectroscopy of PHBV:PLA blends with increasing CB nanofiller percentages at 10${}^{3}$ Hz: (**a**) Resistance versus CB%, (**b**) impedance versus CB%, (**c**) resistance versus PLA fraction, and (**d**) impedance versus PLA fraction.

Impedance (Z) is defined as the effective resistance of a component to an alternating current made up of real (*Z*’) and imaginary (Z”) components (Z=Z’+jZ”). The real and imaginary components of impedance are classified as resistance and reactance respectively. As frequency is increased, *Z*’ will rapidly decrease in insulators and remain constant in conductive materials. Figure 3 shows the resistance (R), and impedance, Z, by weight percent CB (Figure 3a,b) and by volume percent PLA (Figure 3c,d). Figure A3 in Appendix C.1 shows the full spectrum data collected from impedance spectroscopy.

For blends with CB filler content below 18%, there is a significant decrease in Z around frequencies of $10^{4}$ Hz (shown in Figure A3). This frequency is considered the characteristic frequency ($f_{c}$) at which a dependency on frequency forms. Insulators below $f_{c}$ behave independently of frequency until frequency is increased above $f_{c}$ where they become frequency dependent. Samples above 18% CB did not experience this drop off and were considered to behave independent of frequency.

As expected, increasing the percentage of CB added to the matrix increased the conductivity of the nanofilled blend. Using the Sigmoidal-Boltzmann function to fit the resistivity showed that with increasing volume percent of PLA, the percolation threshold ($\varphi_{c}$) shifted from 3.6% in neat PHBV to a minimum of 1.6% for 80:20 PHBV:PLA. Table 5 gives the values for $\varphi_{c}$ with the PLA percentage of the blend. Depending on the conditions for the selective localization of CB at the interface or in one of the polymer phases, in this case PHBV, $\varphi_{c}$ changes with the relative amounts of the polymer phases in the system [59,60]. Other researchers have seen a similar optimization in the mechanical properties for a related 80:20 system [61].

The 80:20 PHBV:PLA 2% BioC sample that was fabricated and measured, had a resistance of 3.43 × 10${}^{8}$ Ω as compared to 2.99 × 10${}^{8}$ Ω for the 2% CB samples, indicating that BioC has the potential to perform similarly to CB when the BioC is produced via the method described herein, provided that challenges during composite fabrication (Section 4.3.1) can be overcome. Converting the measured resistance to resistivity, at 18 wt.% CB the resistivity of the PHBV:PLA nanocomposites ranges from 128 Ω·cm to 167 Ω·cm, which is comparable to commercially available filament (∼0.5–115 Ω·cm) [28,29].

#### 4.2.2. Polymer-Nanofiller Interactions

Raman spectroscopy provided insight both into the graphitic content of the BioC as well as the polymer-nanofiller interface in the blends. Table 6 gives the D and G bands for BioC as compared to previously characterized CB [49]. As expected, for both CB and BioC, only partial graphitization is present, as evidenced by the contribution of the D band which corresponds $sp^{3}$ carbon and is attributed to a higher proportion of defects [50,62]. The intensity ratio between the D and G bands (IDIG) is similar between CB and BioC, potentially indicating similarities in the graphitic and disordered carbon content, although this relationship is complex and is also linked to pyrolysis conditions [63,64,65]. Raman spectroscopy does support the formation of $sp^{2}$ states in the lignin-derived BioC which contribute to electrical conductivity.

Raman spectra provide insight into the polymer-nanofiller interaction via excitation energy shifts upon being incorporated into composites [66]. The Raman spectra corresponding to the D and G bands of both nanofillers were clearly observable in the composites. The CB nanofilled composites had a minimal downshift in the G and D peak intensities upon incorporation of the CB. The BioC showed a more significant upshift in the G band of 39 cm${}^{-1}$. This G-band shift is often observed in chemical modification of the carbon, the presence of electron-donor or acceptor impurities, and surface interactions at the polymer-filler interface [66,67]. Given that there is a complex relationship between the biopolymers and the lignin-derived BioC (Section 4.3), this shift can likely be attributed to that interaction.

#### 4.2.3. Dynamic Mechanical Analysis

**Figure 4:**
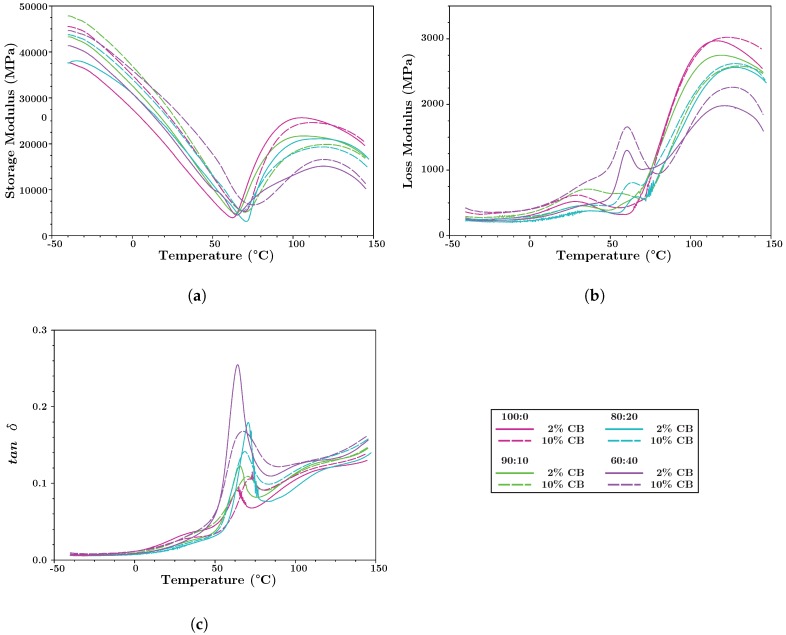
A subset of the DMA data (for 2% and 10% CB) showing: (**a**) Storage modulus, (**b**) loss modulus, and (**c**) loss tangent (tan δ) for PHBV:PLA = 100:0, 90:10, 80:20, and 60:40.

Figure 4 shows the mechanical analysis of a subset of the PHBV:PLA:CB nanocomposites for blends below or near the percolation threshold (2% CB) and above the percolation threshold (10% CB). At low temperatures, below the $T_{g}$ for PLA, there is an increase in elastic modulus of ∼5500 MPa ± 1695 MPa for the high versus low nanofilled composites. This increase is likely due to the combined increase in PHBV and PLA crystallinity between the 2% and 10% CB composites (Section 4.3.2). At high temperatures, the storage modulus of the composites is primarily affected by the blend ratio (PHBV:PLA).

The loss modulus is related to the material’s ability to dissipate mechanical energy and the loss tangent (tanδ), or the ratio between the loss and storage moduli, is related to damping. In composites, these values can be linked to interfacial interactions and toughness [68,69]. Both were influenced by the CB content and blend ratio, with the loss modulus at high temperature increasing slightly in the high nanofilled composites while decreasing with increasing PLA content. There is not a clear dependence of tanδ on CB or blend ratio except for around $T_{g}$. All blends show a prominent peak in tanδ around 65.5 ${}^{\circ }$C ± 2.3 °C for 2% CB and shifted upwards to 70.4 ${}^{\circ }$C ± 1.3 °C for 10% CB. The observed peak is near the $T_{g}$ for PLA, however as even the 100% PHBV composite shows this peak, there is also a contribution due to the presence of the nanofiller. For blends with PLA, the magnitude of the tanδ peak decreased between the low and high CB composites, indicating that the presence of additional nanofiller reduced material damping around $T_{g}$. For all of the blends there was a shift in tanδ towards higher temperatures with increasing CB content, which can be attributed to the increased nanofiller content inhibiting chain movement [70,71]. This shift can also indicate increased thermal stability in the nanofilled composites. The nanofilled composites also improved temperature stability in the loss and storage moduli over that of neat PHBV (data not shown), which started to decrease around 105 °C as compared to ∼120 °C for the nanofilled composites. Like the electrical percolation of the nanofiller through the polymer blends, the increases in storage modulus and tanδ can be linked to the intercalation of the nanofiller through the matrix.

### 4.3. Nanofillers and Fabrication

The behavior of the nanofillers during fabrication was highly dependent on the nanofiller type, CB or BioC. Melt rheology during compounding showed that the melt viscosity of the CB blends increased with increasing nanofiller content, while the viscosity of the BioC blends decreased significantly with the addition of the nanofiller. This effect became more pronounced with increased addition of BioC. DSC showed a modest effect on the melting temperature ($T_{m}$) and crystallinity (χ) upon the addition of CB to the blends. In the case of BioC, the addition of small amounts of nanofiller had a pronounced effect on both $T_{m}$ and χ.

#### 4.3.1. Rheology During Extrusion

**Figure 5:**
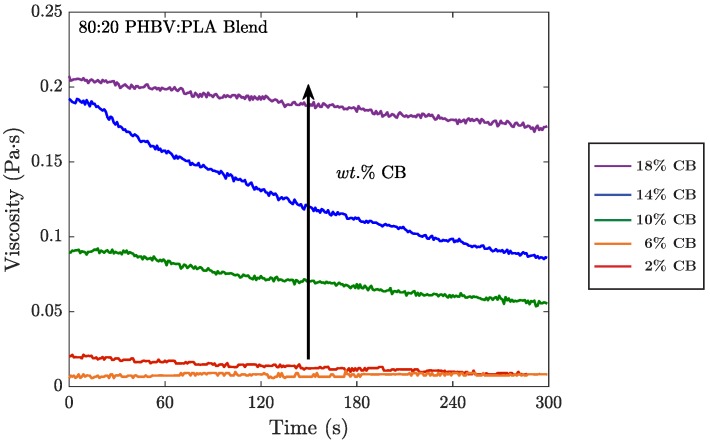
Melt rheology of the 80:20 PHBV:PLA blends during melt blending. Increasing wt.% of CB causes an increase in in the viscosity of blends during mixing.

In addition to modifying the electrical and mechanical properties of the solid composites, the nanofillers had disparate effects on the melt rheology of the blends during mixing. For CB, the rheology measurements during the mixing cycle of the extruder were largely as expected. Early in the mixing cycle, viscosity peaks prior to full mixing of the nanofilled biopolymer blends. As filler and polymers mix, the viscosity of the system begins to decrease until it plateaus. This plateau indicates that the nanofillers are incorporated and melt blending has stabilized, which occurred by 5 min for all CB samples. Also as expected, as CB increased in the blends, the melt became more viscous (Figure 5). This effect is attributed to the added amount of filler in the mixture. Alternatively, the viscosity of BioC blends drops to zero nearly immediately as the filler is added, resulting in a polymer blend that is too fluid for extrusion and injection molding. Due to this rapid reduction in melt viscosity and underlying material causes for the reduced viscosity, BioC nanofilled blends above 2 wt.% could not reproducibly be processed into dimensionally stable composites. The unexpected effect of the BioC on melt viscosity is likely due to chemical reactions with the polymer matrix, as described in Section 4.3.2 and Appendix A.

#### 4.3.2. Differential Scanning Calorimetry

**Figure 6:**
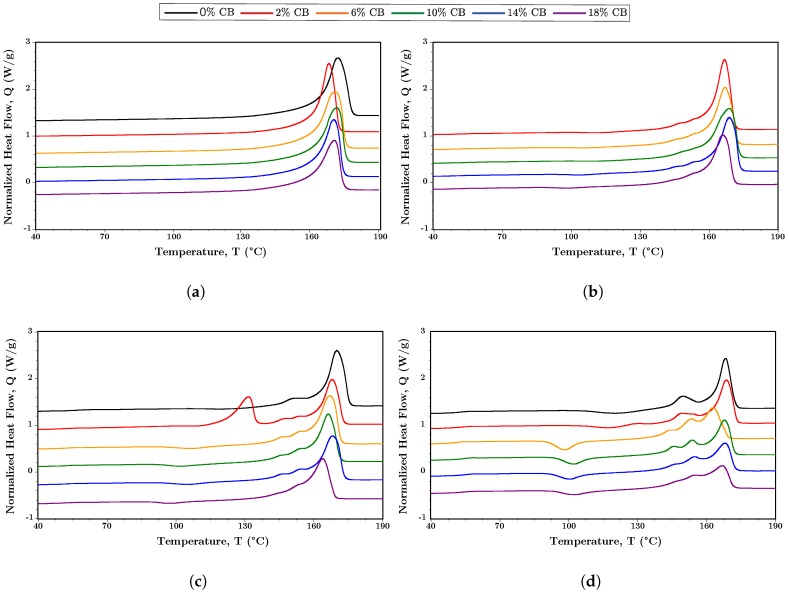
Stacked DSC (endo up) of the melting peak of PHBV:PLA blends with increasing wt.% of CB: (**a**) PHBV:PLA = 100:0, (**b**) PHBV:PLA = 90:10, (**c**) PHBV:PLA = 80:20, and (**d**) PHBV:PLA = 60:40.

**Figure 7:**
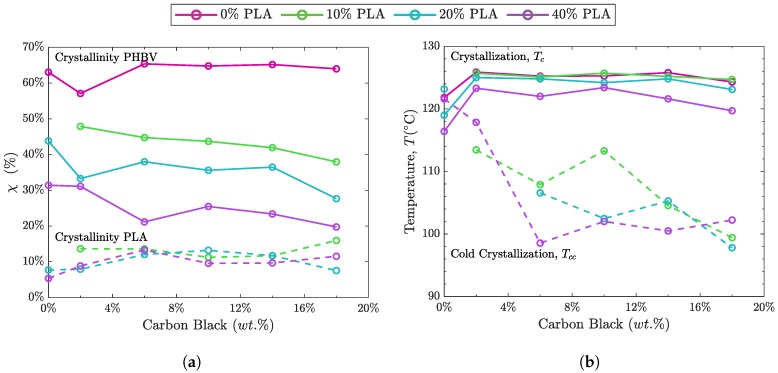
DSC results summarizing: (**a**) The percent crystallinity (χ) as determined through DSC for (solid lines) the PHBV phase of the blend and (dashed lines) the PLA phase of the blend. The impact of the vol.% of the polymer within the blend is more significant in determining crystallinity than the wt.% of CB; (**b**) the shift in crystallization temperature, $T_{c}$, with increasing CB for the PHBV phase of the blend (solid lines) and the shift in cold crystallization temperature, $T_{cc}$, for the PLA phase of the blend (dashed lines). For the PLA phase, $T_{cc}$ decreases with increasing wt.% of CB.

Figure 6 shows the DSC results for the melting peaks of the blended polymers with increasing wt.% CB. In Figure 6a for neat PHBV with CB, the melting peak shifts slightly towards lower temperatures with increasing CB. With increasing PLA content in the blend, Figure 6b–d, the impact on the melting behavior becomes more pronounced. Figure 6d for the blend (black line) clearly displays the split melting peak, PHBV (170.1 °C) and PLA (149.3 °C), anticipated for a phase-separated blend [6,57,69]. With increasing CB, there is a reduction in the main melting peak areas for both components, which results in a decrease in crystallinity, χ, attributed to these primary peaks. This reduction is shown in Figure 7a (solid lines, PLA 10–40%), with the reduction of χ for PHBV. After an initial increase at low CB%, the crystallinity of the PLA phase remains largely unchanged with increasing CB, Figure 7a (dashed lines), due in part to attributing the shoulder peaks to the PLA phase. This increase in peak shoulders and the shift toward lower temperatures (e.g., Figure 6d) is indicative of decreased crystallite size and order likely due either to molecular weight reduction in the PHBV component, PLA component or both, or nanofiller-induced disruption of the crystalline phases [8,43,72].

Another feature, most clearly observable for Figure 6c,d, is the presence of a cold crystallization peak, which is most pronounced for CB wt.% of 10% and above. This peak shows a general trend of shifting towards lower temperature with increased CB (Figure 7b, dashed lines). The presence of cold crystallization indicates barriers to full crystallization during cooling. Because PLA has a cold crystallization peak in the region of the transition observed in the nanofilled composites, we attribute this cold crystallization to the PLA phase (Appendix C.2, Table A1) [73]. The presence of cold crystallization with increased CB supports nanofiller disruption of the crystallization process in the blends. Appendix C.2, Table A1 gives the complete DSC data for the CB nanofilled blends.

In addition to the reduction in crystallinity in the PHBV phase due to the CB content, there is a more pronounced reduction with the increase in PLA content. Also, while the PLA phase shows a reduction in the cold crystallization peak, the crystallization peak of the blend has an initial increase due to the presence of the nanofiller and then remains largely independent of CB content. Therefore, while χ shows some effect due to the nanofiller, the PHBV phase is influenced primarily by the vol.% of PLA. Toughness and physical effects of aging in PHBV:PLA blends have been shown to improve above that of the neat polymers, because of this tailoring of crystallinity and phase interactions, indicating the potential of these blends for enhanced stability and mechanical properties [58,61].

**Figure 8:**
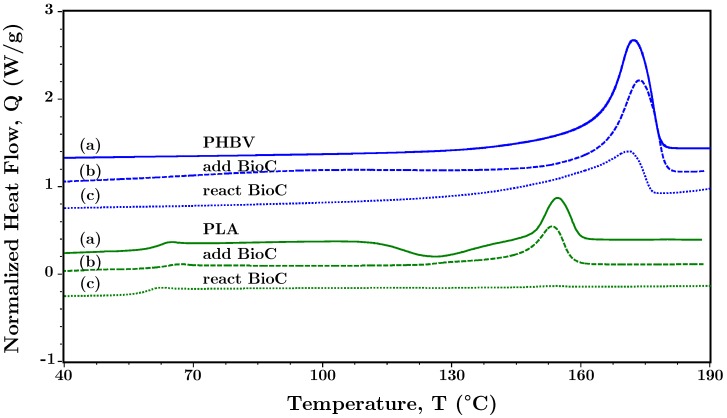
DSC of the melting peak (endo up) of (blue) PHBV and (green) PLA showing (**a**) the neat polymer, (**b**) the neat polymer upon adding BioC in the first heating cycle, and (**c**) the same sample as in b, in the second heating cycle. The effects of mixing BioC with neat polymers is evident in the reduction of both melting peaks, resulting in the absence of a crystalline melting peak for PLA.

**Figure A1:**
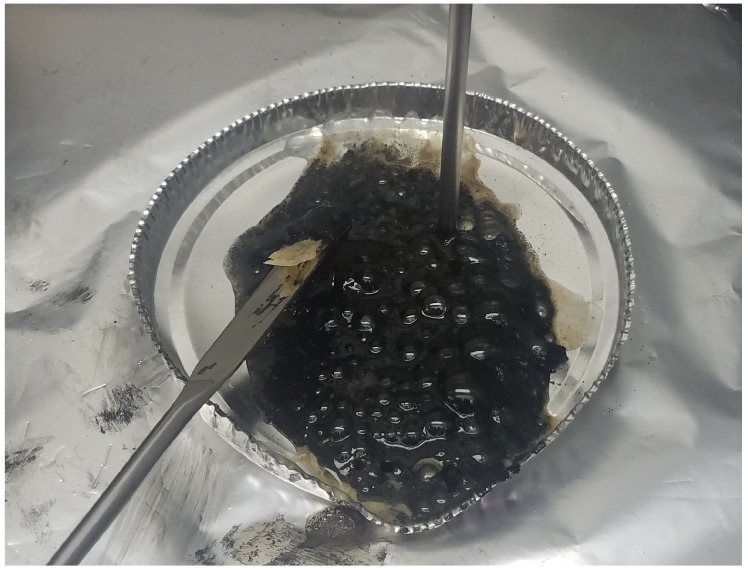
Reaction of 80:20 PHBV:PLA with BioC. Reaction occurs in close proximity to the melting temperature.

**Figure A3:**
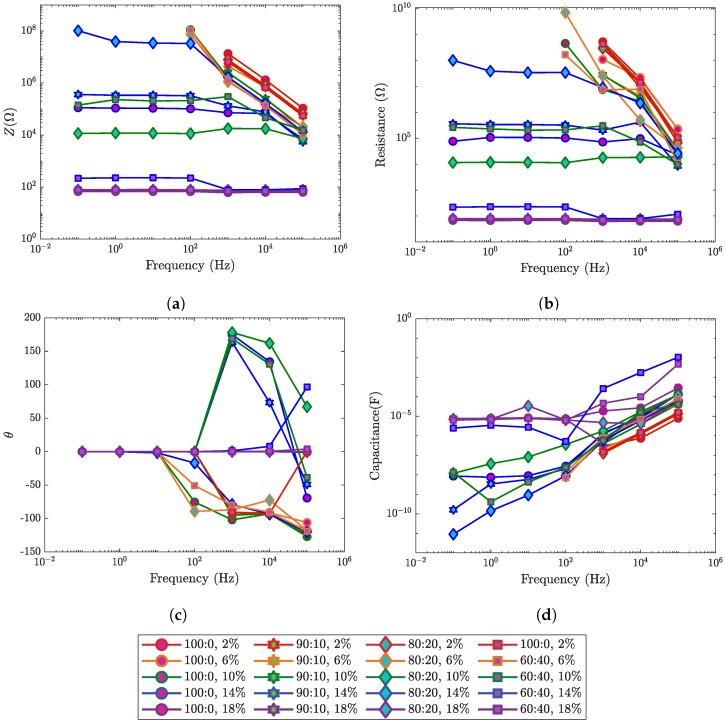
Four point impedance testing of PHBV:PLA blends at increasing CB nanofiller percentages: (**a**) impedance, (**b**) resistance, (**c**) angle, and (**d**) capacitance versus frequency.

In contrast, the effects of adding BioC to the blends were significantly different. As discussed in Section 4.3.1, when added to the melt, the viscosity decreased substantially. To explore this effect, Figure 8 shows a comparison between neat PHBV, neat PLA, and BioC added to PHBV and PLA prior to the heating cycles in the DSC. BioC has a substantial effect on the melting endotherm for both PLA and PHBV. PLA:BioC exhibits a melting peak around 150 °C on the first cycle, as would be expected in neat PLA. PHBV:BioC also has a melting peak similar to the neat polymer on the first cycle. After mixing with BioC and undergoing a thermal cycle, PLA:BioC exhibits no melt peak implying that PLA no longer has crystalline regions. A similar effect takes place in the PHBV:BioC, with a significant reduction in the area of the peak. These results indicate that the significant material changes in the blend upon addition of this lignin-based BioC are likely due to a reaction between the BioC and the polymers. This reaction renders use of BioC prepared from this source difficult for use in extrusion and injection molding. Additional evidence of this reaction is given in Appendix A, Figure A1.

## 5. Conclusions

Conductive nanofiller localization was tailored through phase separation in the PHBV:PLA blends. The partitioning of the nanofiller for the phase-separated morphology of the blends was calculated from surface energies derived from contact angle analysis. FE-SEM was used to verify the location of the nanofillers; CB localized within the PHBV phase as predicted by our calculations. The lignin-derived BioC had a more complex reaction with the biopolymers, as evidenced by the change in blend morphology as observed via FE-SEM, a rapid reduction in melt upon addition of BioC, and DSC results that show a significant reduction in the PHBV melting peak and the absence of a melting peak for the PLA phase. These material changes upon the addition of BioC are likely due to a reaction occurring between the biopolymers and BioC, potentially due to residual species from the Kraft lignin process. The rapid reduction in melt viscosity and source of lignin-BioC/polymer interaction will be critical to implementing BioC as a potential alternative to CB.

The impedance of CB in PHBV:PLA blends was measured for weight percentages of 0, 2, 6, 10, 14, and 18 wt.% CB and PHBV:PLA blends of 100:0, 90:10, 80:20, and 60:40. The phase-separated blends also modified the percolation threshold, which varied between 3.6% and 1.6% CB with the maximum value for 100% PHBV and the minimum for the 80:20 blend. When comparing CB composites with BioC composites with 2% nanofiller (approximately at the percolation threshold), the 2% BioC had a resistance of 3.43 × 10${}^{8}$ Ω as compared to 2.99 × 10${}^{8}$ Ω for the CB, indicating that BioC could perform comparably to CB as a conductive nanofiller if the processing challenges can be overcome. Results at higher BioC loading would be required to establish if this comparable behavior is present for more conductive samples. Both nanofillers exhibited graphitic content individually and incorporated into PHBV:PLA blends, as determined via Raman spectroscopy, which is necessary for a conductive network of nanoparticles. These results further support the use of nano sized lignin-derived BioC as an electrically conductive nanofiller in biocomposites.

## Figures and Tables

**Table 1:** PHBV:PLA composite blend ratios with CB and BioC nanofiller.

	Vol.% of Blend					wt.% of Total				
PHBV	100	90	80	60	Nanofiller (CB, BioC)	2	6	10	14	18
PLA	0	10	20	40	PHBV:PLA blend	98	94	90	86	82

**Table 2:** Surface tensions for water and diiomethane.

Liquid	γ	$\gamma^{p}$	$\gamma^{d}$
Water	72.8	51.0	21.8
MI	50.8	0.4	50.4

**Table 3:** Contact angles and surface tensions using the Owens-Wendt model.

	Contact Angles		Surface Tensions			
	θ **H** ${}_{2}$ **O**	θ **MI**	γ **(20** ° **C)**	**γ (190 °C)**	$\gamma^{d}$	$\gamma^{p}$
	**deg** °	**deg** °	**(mJ/m**${}^{2}$)	**(mJ/m**${}^{2}$)	**(mJ/m**${}^{2}$)	**(mJ/m**${}^{2}$)
PHBV	64.55 ± (1.06)	47.24 ± (0.98)	45.095	35.195	32.365	12.729
PLA	64.00 ± (0.66)	62.02 ± (3.05)	40.996	31.096	23.892	17.104
CB [41,42]	-	-	98.1	87.9	84.1	3.2

**Table 4:** Interfacial tension and spreading coefficients between polymers and nanofiller.

Component	Interfacial Tension	Blend (i:j)	Spreading	$\theta_{\mathrm{ij}}^{o}$	Wetting	Particulate
	$\frac{\mathrm{mN}}{m}$		**Coef**		**Coef**	**Localization**
PHBV/PLA	1.924	PHBV:PLA	−6.0072	29.918	−7.4971	PHBV
PHBV/CB	5.187	PLA:PHBV	2.1592	150.08	7.4971	PHBV
PLA/CB	8.682					

**Table 5:** Percolation threshold, $\varphi_{c}$, with volume percent PLA.

PHBV:PLA	$\varphi_{c}$
**vol.**%	**wt.% CB**
100:0	3.6 ± 0.02
90:10	2.4 ± 0.90
80:20	1.6 ± 0.60
60:40	2.4 ± 0.01

**Table 6:** Graphitic (G) and disordered (D) bands and peak intensity ratio (IDIG) in CB and BioC nanofilled blends.

	D	G	IDIG
	(1320–1370 cm${}^{-1}$)	(1530–1610 cm${}^{-1}$)	
CB [49]	1359	1604	1.12
BioC	1366	1534	1.13
80:20, 6% CB	1342	1599	1.08
80:20, 6% BioC	1368	1573	1.03

**Figure A2:**
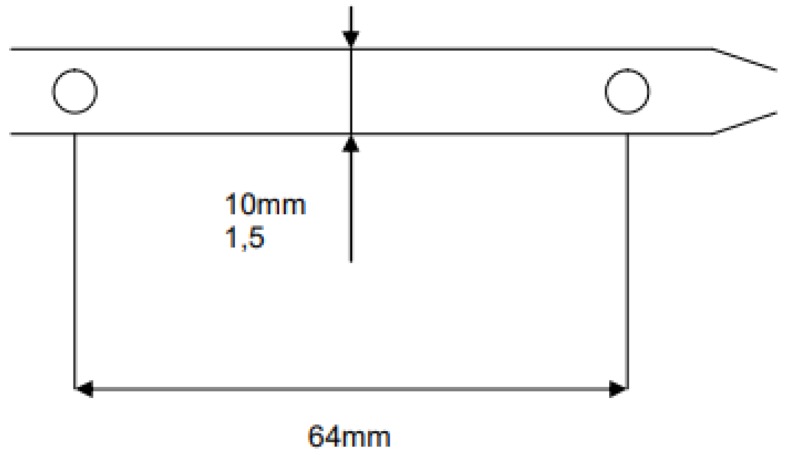
Flow channel of the HAAKE Minilab, provided by Thermo Fisher Scientific. The circles denote the pressure transducers.

## Appendix A. BioC Modifications to Melt Viscosity

In order to investigate the large reduction in melt viscosity with increasing addition of BioC, we heated a blend of 80:20 PHBV:PLA with BioC on a hot plate at a controlled temperature ramp. Figure A1 shows the visual results of this experiment. Upon initial melting there was an apparent reaction with bubbling and rapid liquefaction of the melt as compared to the blend without BioC. These observations prompted experiments with DSC (Appendix C.2, Table A1).

## Appendix B. Supplemental Processing Methods

In order to accurately calibrate the shear rate ($\gamma^{*}$) and viscosity ($\eta^{*}$) during mixing, we measured the volumetric flow rate of our primary phase, PHBV, and applied mixing chamber geometry provided by Thermo Fisher Scientific for the HAAKE Minilab II to calculate $\gamma^{*}$ and $\eta^{*}$. The Minilab is equipped with a back flow channel designed as a slit capillary with a pressure transducer in the capillary entrance and one pressure transducer at the capillary exit (Figure A2).

- Distance between the transducers: ΔL=64 mm
- Depth of the flow channel: h=1.5 mm
- Width of the flow channel: w=10 mm

The pressure transducers measure the pressure drop in the capillary. From the capillary geometry and the pressure drop the shear stress (τ) is calculated:

(A1) $$\tau =\left(\frac{h}{2\Delta L}\right)\Delta P=\left(0.01171875\right)\Delta P$$

where *h* and ΔL are defined as above, and ΔP is the pressure change between the two pressure transducers. Because the Minilab does not measure the absolute volume of the flow, the values for $\gamma^{*}$ and $\eta^{*}$ are calculated from the apparent flow volume through the capillary. This apparent flow volume, $\dot{V}$, is proportional to the screw speed, *n*:

(A2) $$\dot{V}=C*n$$

where the correlation factor, *C*, was determined experimentally for PHBV by measuring mass flow rate out of the extruder: *C* = 2 × 10${}^{-7}$. From the volume flow $\dot{V}$ and the capillary geometry, the apparent shear rate ($\gamma^{*}$) was calculated:

(A3) $$\gamma^{*}=\left(\frac{6}{wh^{2}}\right)=\left(2.\bar{6}x10^{8}\right)\dot{V}$$

and from this result the viscosity ($\eta^{*}$) was calculated:

(A4) $$\eta^{*}=\frac{\tau }{\gamma^{*}}=\frac{wh^{3}}{12*\Delta L}\frac{\Delta P}{\dot{V}}=\left(4.3945x10^{11}\right)\left(\frac{\Delta P}{\dot{V}}\right)$$

## Appendix C. Supplemental Data

### Appendix C.1. Impedance Measurements for All Frequencies

Figure A3 shows the data for the PHBV:PLA blends at CB percentages of 2%, 6%, 10%, 14%, and 18%.

### Appendix C.2. Differential Scanning Calorimetry

Table A1 gives the thermal transition temperatures and enthalpies for the complete set of PHBV:PLA:CB blends. As observed in our work and prior studies of blends [57], the $\Delta H_{m}$ of the individual components of the blends can be measured by splitting the melt peak (endothermic) into two main regions and assuming the exothermic $\Delta H_{cc}$ peak due to cold crystallization can be attributed to the PLA phase. The resulting crystallinity for each phase of the blend was calculated as described in Section 3.4.3.

**Table A1:** Differential scanning calorimetry of the PHBV:PLA blended polymers.

Composition	Filler	$T_{g}$	$T_{\mathrm{cc}}$	$T_{c}$ PHBV:PLA	$T_{m}$	$\Delta H_{m}$	$\Delta H_{\mathrm{cc}}$	$X_{c}$
	wt.% CB	°C	°C	°C	°C	J/g	J/g	%
100:0	0	-	-	122	172	92.0	-	63
100:0	2	-	-	126	169	81.7	-	57
100:0	6	-	-	125	171	89.7	-	65
100:0	10	-	-	125	172	85.1	-	65
100:0	14	-	-	126	171	81.8	-	65
100:0	18	-	-	124	171	76.6	-	64
PLA Component								
90:10	2	51.97	113.44	-	152.00	16.24	3.47	13.6
90:10	8	51.36	107.90	-	152.00	16.11	3.38	13.6
90:10	10	54.12	113.29	-	152.00	14.00	3.46	11.2
90:10	14	51.69	104.55	-	152.00	12.70	1.76	11.7
90:10	18	51.28	99.41	-	152.00	16.73	1.79	15.9
80:20	0	57.21	123.17	-	150.00	10.52	3.31	7.7
80:20	2	52.30	0.00	-	153.00	10.46	0.00	7.9
80:20	8	53.19	106.57	-	153.00	14.87	3.04	12.0
80:20	10	53.44	102.46	-	153.00	14.83	3.60	13.2
80:20	14	53.30	105.28	-	153.00	12.56	2.47	11.7
80:20	18	52.18	97.80	-	153.00	16.65	1.60	7.5
60:40	0	52.37	121.60	-	149.00	14.59	9.59	5.3
60:40	2	52.65	117.85	-	153.00	14.06	5.79	8.8
60:40	8	50.66	98.54	-	153.00	21.85	9.39	13.3
60:40	10	53.76	102.00	-	153.00	16.01	7.05	9.6
60:40	14	54.58	100.48	-	155.00	15.30	6.26	9.7
60:40	18	53.74	102.22	-	155.00	14.88	4.08	11.5
PHBV Component								
90:10	2	-	-	125.74	166.88	69.88	-	47.87
90:10	8	-	-	125.13	167.04	65.32	-	44.74
90:10	10	-	-	125.71	169.04	63.78	-	43.69
90:10	14	-	-	125.24	169.08	61.20	-	41.92
90:10	18	-	-	124.74	166.15	55.40	-	37.95
80:20	0	-	-	119.03	170.08	63.96	-	43.81
80:20	2	-	-	125.00	168.03	48.57	-	33.27
80:20	8	-	-	124.83	167.17	55.44	-	37.98
80:20	10	-	-	124.18	166.39	51.97	-	35.60
80:20	14	-	-	124.84	168.13	53.26	-	36.48
80:20	18	-	-	123.14	163.81	40.38	-	27.66
60:40	0	-	-	116.37	168.42	45.85	-	31.40
60:40	2	-	-	123.30	168.72	45.40	-	31.10
60:40	8	-	-	121.96	163.05	30.89	-	21.16
60:40	10	-	-	123.40	167.79	37.18	-	25.47
60:40	14	-	-	121.60	168.12	34.13	-	23.38
60:40	18	-	-	119.65	166.85	28.82	-	19.74
